# What Do We Really Know About 5-HT_1A_ Receptor Signaling in Neuronal Cells?

**DOI:** 10.3389/fncel.2016.00272

**Published:** 2016-11-24

**Authors:** Paulina S. Rojas, Jenny L. Fiedler

**Affiliations:** ^1^Laboratory of Neuroplasticity and Neurogenetics, Department of Biochemistry and Molecular Biology, Faculty of Chemistry and Pharmaceutical Sciences, Universidad de ChileSantiago, Chile; ^2^Faculty of Medicine, School of Pharmacy, Universidad Andres BelloSantiago, Chile

**Keywords:** serotonin, 5HT_1A_R, signaling, CHO, neurons, ERK, AKT, cytoskeleton

## Abstract

Serotonin (5-HT) is a neurotransmitter that plays an important role in neuronal plasticity. Variations in the levels of 5-HT at the synaptic cleft, expression or dysfunction of 5-HT receptors may alter brain development and predispose to various mental diseases. Here, we review the transduction pathways described in various cell types transfected with recombinant 5-HT_1A_ receptor (5-HT_1A_R), specially contrasting with those findings obtained in neuronal cells. The 5-HT_1A_R is detected in early stages of neural development and is located in the soma, dendrites and spines of hippocampal neurons. The 5-HT_1A_R differs from other 5-HT receptors because it is coupled to different pathways, depending on the targeted cell. The signaling pathway associated with this receptor is determined by G_α_ isoforms and some cascades involve βγ signaling. The activity of 5-HT_1A_R usually promotes a reduction in neuronal excitability and firing, provokes a variation in cAMP and Ca^2+^, levels which may be linked to specific types of behavior and cognition. Furthermore, evidence indicates that 5-HT_1A_R induces neuritogesis and synapse formation, probably by modulation of the neuronal cytoskeleton through MAPK and phosphoinositide-3-kinase (PI3K)-Akt signaling pathways. Advances in understanding the actions of 5-HT_1A_R and its association with different signaling pathways in the central nervous system will reveal their pivotal role in health and disease.

## Introduction

Serotonin (5-HT) is a chemical mediator, synthesized from tryptophan, that has been maintained throughout evolution. In mammals, in addition to its role as a neurotransmitter, 5-HT has been described as a regulator of neuronal connectivity during development by modulating cell migration and cytoarchitecture (Lauder, 1993). Indeed, abnormal levels of 5-HT result in aberrant morphology and wiring of the nervous system in mammals (for review see Gaspar et al., 2003). Alterations in neural circuits observed in adults may be related to dysfunction in the actions and/or levels of 5-HT during key stages of development, which may predispose juvenile and adult individuals to various mental diseases (Hornung, 2003). Thus, a number of factors that can modify 5-HT levels during pregnancy may alter brain development: changes in nutrition affecting the availability of tryptophan (Serfaty et al., 2008), challenges to stressors (Papaioannou et al., 2002), infections (Winter et al., 2009) and antidepressant drugs that act as serotonin reuptake inhibitors (SSRIs; Xu et al., 2004).

The serotonin receptors have been classified as 5-HT_1A-F_, 5-HT_2A-C_; 5-HT_3_, 5-HT_4_, 5-HT_5_, 5-HT_6_ and 5-HT_7_. Unlike the 5-HT_3_ receptor that is ionotropic (Mattson et al., 2004), the remaining receptors are coupled to different G proteins (Albert and Tiberi, 2001). Given the diversity of 5-HT receptors, it has been difficult to define their precise role on brain development, either individually or in combination with other receptors. Nonetheless, immunohistochemical studies show that these receptors are expressed early during embryonic development and are dynamically regulated postnatally, suggesting a pivotal role during brain development (Gaspar et al., 2003). In the present article, we will extensively review the existing literature on 5-HT_1A_ receptor (5-HT_1A_R)-mediated signaling in neurons, mainly in the hippocampus brain area. It is important to highlight that many of the signaling pathways associated with the 5-HT_1A_R have been derived from studies in non-neuronal cells, revealing the important contribution of this review on the neuroscience field.

## 5-HT_1A_R Distribution in the Hippocampus during Development and Adulthood

The 5-HT_1A_R transcript is detected in the rodent fetal brain at stage E12, achieves a maximum level at E15 and then progressively reduces its expression to low levels before birth (E20; Hillion et al., 1993). The expression of 5-HT_1A_R coincides with the migration of young neurons to their appropriate neuronal stratum during embryonic development (Patel and Zhou, 2005). In the hippocampus, neurons begin to express the 5-HT_1A_R at around E16; just 1–2 days after mitosis accomplishment and prior to migration to the laminar layer (Patel and Zhou, 2005). In developing hippocampus at E18, this receptor is detected in interneurons located in *stratum radiatum* and *stratum oriens* (Patel and Zhou, 2005). Furthermore, 5-HT_1A_R is also detected in the soma and emerging neurites of young neurons, which have just reached the *stratum pyramidale* (Patel and Zhou, 2005). We have recently detected 5-HT_1A_R mRNA and protein at 2 and 3 days *in vitro* (DIV) in hippocampal primary cultures obtained from E18 fetuses (Rojas et al., 2014). In addition, during postnatal development, the 5-HT_1A_R is redistributed from the soma to the basal and apical dendrites; a phenomenon observed in both pyramidal and granule neurons of the hippocampus (Patel and Zhou, 2005). Interestingly, in brain neurons, the Ypt1p interacting factor homolog B (Yif1B) has been identified as a vesicular membrane-bound scaffolding protein which interacts directly with the C-terminal domain of the rat 5-HT_1A_R to mediate the intracellular trafficking of this receptor towards dendrites (Carrel et al., 2008). Additionally, the somato-dendritic distribution of 5-HT_1A_R detected early in the hippocampus prevails in adult animals; also displaying a location at dendritic spines (Riad et al., 2000). Furthermore, the somatic-dendritic redistribution of this receptor may be associated with the differential actions of 5-HT; i.e., in the soma, receptor activation may be associated with the regulation of cell growth by controlling gene expression and neuronal excitability; but in dendrites, this receptor may regulate neuronal morphology (Patel and Zhou, 2005). In adult animals, interestingly, the 5-HT_1A_R is detected in the subgranular layer of the dentate gyrus and its activation increases the proliferation of granule cell precursors in this hippocampal area (Gould, 1999).

## 5-HT_1A_R Activation Modulates Neuronal Excitability and Responsiveness to Neurotransmitters

In both neurons and brain tissue, few signal transduction cascades associated with the activity of the 5-HT receptors have been described. Serotonergic fibers spread diffusely in brain and often lack direct synaptic contacts; however the release of 5-HT may play an important role in the fine tuning of neuronal communication in the hippocampus (Vizi and Kiss, 1998). The activity of the 5-HT_1A_R allows a modulatory effect by changing neuronal firing. Electrophysiological studies have shown that stimulation of 5-HT_1A_R in serotonergic neurons of the raphe nuclei (autoreceptor) induces cell hyperpolarization and a reduction in 5-HT release (Polter and Li, 2010). Furthermore, the activation of 5-HT_1A_R exerts hyperpolarizing effects in hippocampal neurons (Dong et al., 1997; Salgado-Commissariat and Alkadhi, 1997; Tokarski et al., 2002; Tada et al., 2004). Nonetheless, in ventral hippocampus, 5-HT_1A_R activity produces an indirect excitatory response through the inhibition of GABAergic interneuron activity induced by hyperpolarization (Schmitz et al., 1995b).

**Figure 1:**
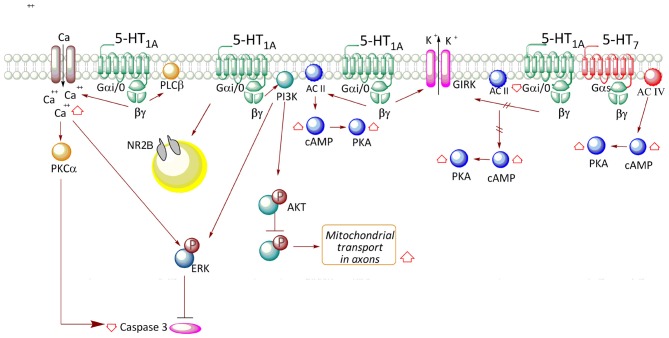
**Transductional pathways associated with 5-HT_1A_ receptor (5-HT_1A_R) activation in neuronal and neuronal cell lines.** In neurons, receptor activation releases βγ and promotes an increase in AC II activity, with concomitant increase in AMPc levels and PKA activation. The βγ complex also participates in the activation of the phosphoinositide-3-kinase (PI3K)-Akt pathway, which triggers an increase in phospho-ERK levels. Moreover, the PI3K-Akt-GSK-3β pathway increases mitochondrial transport in axons. Additionally, stimulation of the receptor increases Ca^2+^ levels, which also contributes to the activation of PKCα and ERK, reducing caspase-3 levels. Release of the βγ complex also activates a K^+^ rectifier channel (GIRK), allowing cell hyperpolarization. According to that described in cell lines, the association between receptor activity and the reduction in AC I activity is only valid in the case of the autoreceptor, such as in neurons of the raphe nucleus.

On the other hand, glutamate receptor-mediated transmission between CA3 and CA1 pyramidal neurons can be depressed by 5-HT_1A_R activity (Costa et al., 2012). The change in cell polarity mediated by 5-HT_1A_R occurs by activation of Gα*_i/o_* and subsequent release of the βγ complex, which triggers the gating of inward rectifying potassium channels (GIRK; Figure 1). Interestingly, in contrast to the desensitization of 5-HT_1A_ autoreceptors (Riad et al., 2001), the persistent activation of 5-HT_1A_Rs coupled to GIRK in the hippocampus does not promote its internalization (Dong et al., 1998). According to this evidence, it seems that the desensitization of 5-HT_1A_Rs depends on the cell type in which the receptors are expressed. Furthermore, it was described that 5-HT_1A_R might reduce excitatory transmission in rat CA1 hippocampal area by a putative presynaptic mechanism that reduces Ca^2+^ entry and glutamate release (Schmitz et al., 1995a).

## 5-HT_1A_ Receptor Activation Mediates Opposing Effects on Adenylate Cyclase Activity in Non-Neuronal and Neuronal Cells

**Figure 2:**
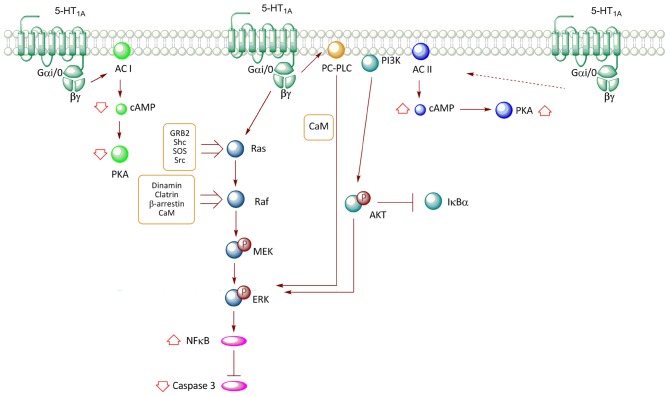
**Transductional pathways associated with the activation of the 5-HT_1A_R overexpressed in non-neuronal cell lines.** Signaling pathways of the 5-HT_1A_-R in CHO (cells derived from Chinese hamster ovary) and HEK293 (human embryonic kidney) cells are described. Activation of the receptor reduces cAMP levels through the inhibition of AC I, with a subsequent decrease in PKA activity; an effect mediated by G*_αi/0_*. In contrast, co-expression of the receptor with AC II promotes an increase in the activity of this enzyme, increasing cAMP levels and PKA activation; effect mediated by βγ. The release of βγ after the activation of the receptor promotes ERK phosphorylation through two pathways, which involve the Ras-Raf-MEK and phosphatidylcholine-specific phospholipase C (PC-PLC) proteins. Moreover, the increase in ERK phosphorylation after the activation of the receptor promotes a reduction in caspase-3 activity; an effect mediated by the activation of the nuclear factor κB (NF-κB) transcription factor. Additionally, the activation of the 5-HT_1A_R also activates the PI3K-Akt pathway, which participates in ERK phosphorylation.

The use of transfection techniques of the human 5-HT_1A_R in different cell lines has allowed further insight about the association of this receptor with specific G protein transducers, and related signaling pathways. In the HEK293 cell line, the activation of 5-HT_1A_R activates Gα_i/o_, leading to a reduction in cAMP levels through inhibition of adenylyl cyclase (AC) type I (Albert et al., 1999; Figure 2). However, when HEK293 cells were co-transfected with the 5-HT_1A_R along with AC type II, the agonist (8OH-DPAT) increased cAMP levels, an effect mediated by the G_βγ_ complex, which stimulates enzyme activity (Albert et al., 1999). Similar effects were observed in co-transfection experiments with pituitary cell lines (Liu et al., 1999). Interestingly, co-transfection with AC type II and Gα_i2_, but not Gα_i1_, Gα_i3_, or Gα_o_, resulted in an agonist-independent increase in basal cAMP levels, suggesting that the Gα_i2_ isoform promotes constitutive activation of the receptor (Albert et al., 1999). In contrast, the presence of both Gα_i2_ and Gα_i3_ results in reduced cAMP levels, suggesting that the action of Gα_i3_ predominates over that of Gα_i2_ (Liu et al., 1999; Figure 2).

*In vivo* microdialysis experiments have shown that systemic administration of 8OH-DPAT, an agonist that displays high affinity for 5-HT_1A_R (0.65 nM) in comparison to 5-HT_7_R (35 nM; Sprouse et al., 2004), increases the efflux of cAMP in the ventral hippocampus (Cadogan et al., 1994). The interpretation of this *in vivo* study is highly complex because the systemic administration of 8OH-DPAT may involve the participation of 5-HT_1A_R located in serotonergic neurons of the raphe nucleus (autoreceptors), which may diminish the liberation of 5-HT in targeted areas. Thus, a reduction of 5-HT_1A_R activity in several structures, including the hippocampus, may occur associated to reduced αi coupling to AC type I, with the consequent enhancement in cAMP effux (Figure 1). On the other hand, it is probable that 8OH-DPAT not only involves 5-HT_1A_R, but also the 5-HT_7_R, which activates AC (Ruat et al., 1993). Nonetheless, the study of Cadogan et al. (1994) also showed that cAMP efflux induced by 8OH-DPAT is blocked by pre-treatment with WAY-100135, an antagonist with high selectivity for 5-HT_1A_R (IC50 = 15 nM) over 5-HT_1B, 1C_, α1 and α2 adrenoceptor and D_2_ receptors (IC50 > 1000 nM; Fletcher et al., 1993). On the other hand, some direct determinations of 5-HT_1A_R activity have been conducted in mammalian guinea pig and rat hippocampal membranes. These studies revealed that 5-HT and 8OH-DPAT stimulate the production of cAMP, although the latter compound showed a reduced efficacy, suggesting the contribution of other receptors such as 5HT_7_R (De Vivo and Maayani, 1986). In contrast, the same study demonstrated that 8OH-DPAT reduces Forskolin-stimulated cAMP production through a receptor with pharmacological characteristics of 5-HT_1A_R (De Vivo and Maayani, 1986). Furthermore, prolonged exposure of cultured hippocampal neurons to 8OH-DPAT did not significantly affect 5-HT_1A_R-induced inhibition of cAMP production, indicating that this receptor does not desensitize in this model (Varrault et al., 1991).

According to discussed evidences, the signaling pathway associated with 5-HT_1A_R is probably determined by the precise Gα isoform existing in cells, even though the presence of other G protein transducers may redirect signal transduction to other existing pathways. Furthermore, considering that AC type II is highly expressed in soma and dendrites of hippocampal neurons (Baker et al., 1999), it is feasible that in restricted areas of hippocampus, the 5-HT_1A_R activates AC type II through the Gβγ complex (Figure 1), similarly to the transfected HEK cell (Figure 2).

## 5-HT_1A_R and MAPK Activation Occurs Through Intricate Pathways in Non-Neuronal Cell Models

Studies in Chinese hamster ovary (CHO) cells transfected with the human 5-HT_1A_R have demonstrated that stimulation with 5-HT and the 5-HT_1A_R agonist, 8OH-DPAT, promotes the phosphorylation of ERK (Cowen et al., 1996; Hsiung et al., 2005). This response was shown to be blocked by pertussis toxin and thus, corroborated the participation of Gα_i_ and Gα_o_ (Cowen et al., 1996; Garnovskaya et al., 1996; Hsiung et al., 2005). 5-HT_1A_-mediated MAPK activation in CHO cells is blocked by specific 5-HT_1A_R antagonists (Cowen et al., 1996; Errico et al., 2001) or dominant negative mutants of GRK, β-arrestin and dynamin; proteins involved in agonist-induced receptor endocytosis (Della Rocca et al., 1999). Additionally, in CHO-1A-27, the increase in phospho-ERK1/2 levels induced by 5-HT is prevented by the addition of an intracellular calcium chelator (BAPTA) and by phenothiazine, an inhibitor of calmodulin (CaM), revealing the participation of Ca^2+^/CaM (Della Rocca et al., 1999; Figure 2). Furthermore, ERK1/2 activation is sensitive to the inhibition of Src type kinases (Garnovskaya et al., 1998). In CHO cells, ERK activation mediated by 5-HT_1A_R involves βγ subunit as transducers (Garnovskaya et al., 1996). The release of βγ subunits induced by 5-HT_1A_R activity triggers the formation of a multimolecular complex, including Grb2, p46Shc, p52Shc, which is required for activation of the exchange factor Son-of-sevenless (SOS), which in turn activates the Ras/Raf/MEK pathway (Garnovskaya et al., 1996; Figure 2). Likewise, inhibition of CaM reduces the activity of both Src tyrosine kinase and the small GTP-ase Ras, but not of Raf kinase and mitogen-activated protein kinase (MEK; Della Rocca et al., 1999). These evidences suggest that the Ca^2+^/CaM complex is required downstream of Ras activation, but upstream of Raf and MEK activation (Della Rocca et al., 1999; Figure 2). It has been established that the third loop of the 5-HT_1A_R contains two binding sites for CaM (Turner et al., 2004); interaction that in HEK293 cells, mediates CaM-induced clathrin-mediated endocytosis of 5-HT_1A_R, a step in the activation of MEK and ERK (Della Rocca et al., 1999; Figure 2). Thus, the mechanism by which 5-HT_1A_R activates the RAS–MAPK pathway through G_βγ_ is still uncertain; it seems to involve recruitment of GRK to phosphorylate the receptor, and both β-arrestin-mediated internalization and Src-like kinases activation upon receptor internalization.

In CHO cells, 5-HT_1A_R-induced activation of ERK involves the participation of phosphatidylcholine-specific phospholipase C (PC-PLC) and phosphoinositide-3-kinase (PI3K; Cowen et al., 1996; Garnovskaya et al., 1996, 1998; Hsiung et al., 2005). In this same cell type, studies have indicated that 5-HT_1A_R agonists prevent activation of caspase-3 induced by serum deprivation, phenomenon associated with the activation of PI3K-PKB (Akt) and ERK pathways (Hsiung et al., 2005; Figure 2). Furthermore, this same study showed that PI3K-Akt activity promotes degradation of IKBα, a protein that inhibits Nuclear Factor κB (NF-κB) transcriptional activity by its retention in the cytosol, with the subsequent NF-κB translocation to the nucleus (Hsiung et al., 2005; Figure 2).

## 5-HT_1A_R and MAPK Engagement in Neuronal Cells: Possible Implication in Neuronal Morphology

Studies performed in the HN2-5 immortalized hippocampal cell line, which overexpresses the 5-HT_1A_R, indicated that stimulation with 8OH-DPAT slowly increases the phosphorylation of ERK, through a mechanism that involves G_αi/o_ protein and PI3K activation (Adayev et al., 1999; Figure 1). Additionally, in HN2-5 cells the 5-HT_1A_R activates PLCβ and increases Ca^2+^ levels, leading to PKCα and ERK activation and inhibition of caspase-3 activation and apoptosis (Adayev et al., 1999, 2003; Figure 1).

Activation of ERK1/2 and the PI3K/PKB signaling pathways not only regulate neuronal differentiation and survival, but also control neurite outgrowth and branching by modulating the reorganization of the cytoskeleton (Kim et al., 2004; Jaworski et al., 2005; Kumar et al., 2005). Some studies have shown that 5-HT depletion in the early postnatal period (P3) causes a reduction in dendrite length and spine density of hippocampal granule neurons and these effects are prevented by the administration of a 5-HT_1A_R agonist (Yan et al., 1997). In line with these results, stimulation of the hippocampal 5-HT_1A_R in organotypic cultures of hippocampi from mice at postnatal period (P15)—that coincides with the peak of synaptogenesis—increases dendritic spine density and synapse formation through sequential activation of ERK1/2 and PKC (Mogha et al., 2012); however, the precise mechanism has not been characterized. *In vitro* studies have indicated that 5-HT_1A_R activation induces an increase in both the number and length of neurites in mouse neuroblastoma (Fricker et al., 2005). Our recently published study using rat hippocampal primary cultures demonstrated that stimulation of 5-HT_1A_R at 2 DIV promotes the growth of secondary neurites (Rojas et al., 2014). The molecular mechanisms underlying the regulation of neurite outgrowth mediated by 5-HT_1A_R remains to be elucidated.

Besides, *in vivo* pharmacological blockade of 5-HT_1A_R with WAY-100635 during 3–5 weeks of postnatal development, significantly increases branch points of the apical dendritic tree in CA1 neurons (Ferreira et al., 2010). Additionally, in a primary culture of mouse hippocampus (5 DIV), stimulation with 5-HT was described to promote depolymerization of filamentous actin in cone growth, an effect observed in WT mice, but not in KO mice for 5-HT_1A_R (Ferreira et al., 2010). Therefore, it has been suggested that 5-HT_1A_R regulates actin dynamics and restricts dendritic growth and thus, modulates neuronal connectivity during a certain period of development (Ferreira et al., 2010). Considering the evidence as a whole, 5HT_1A_R promotes synapse formation but restricts dendrite arborization.

## Activation of the 5-HT_1A_R in Non-Neuronal and Neuronal Cells and Its Relationship with the PI3K-AKT-GSK-3β Pathway

Systemic administration of 8OH-DPAT in mice increases phosphorylation at Thr^308^ and in a lesser degree, Ser^473^ of Akt in hippocampus (Polter et al., 2012). These changes were correlated with an increase in the inactivating phosphorylation of GSK-3β (^9^Ser; Leemhuis et al., 2004; Polter and Li, 2011), effects which are attenuated by the specific 5-HT_1A_R antagonist, WAY-100635. The interpretation of *in vivo* studies is complicated because the systemic administration may involve both the activation of autoreceptors located on serotonergic neurons in the raphe nucleus, or heteroreceptors in other structures different from that of the hippocampus. Therefore, it is possible that changes in the phosphorylation of GSK-3β are product of the contribution of indirect effects of 5-HT receptors located in different brain areas. Interestingly, GSK-3β activity regulates the activity of several microtubule-associated proteins (MAPs) and during development, may direct axon growth and guidance, a process which requires microtubule dynamics (Garrido et al., 2007). The causal link between the activation of 5-HT_1A_R and phosphorylation of Akt and GSK-3β has not been fully documented in cultured neurons. In hippocampal neurons of 5–7 DIV, 5CT, 8OH-DPAT and 5-HT increase phosphorylation of Akt at Ser^473^ (Cowen et al., 2005). Additionally, in a more mature hippocampal culture (17 DIV), stimulation with 5-HT or 8OH-DPAT increases phosphorylation of Akt at Ser^473^, and rises phospho-GSK3β (Chen et al., 2007). Interestingly, 5-HT_1A_R has been reported to promote mitochondrial movement in axons of hippocampal neurons at 17 DIV, and this effect is mediated by the inhibition of GSK-3β promoted by Akt (Chen et al., 2007; Figure 1).

Although the previous evidences indicate a relationship between the activation of 5-HT_1A_R and Akt phosphorylation, it is still unclear whether this depends on the activity of PI3K in a similar manner to that described in CHO cells (Hsiung et al., 2005; Figure 2). However, in hippocampal tissue, the 5-HT_1A_R transduces via G*_αi/0_* and therefore, it is likely that the βγ complex not only regulates neuronal activity through GIRK, but also activates PI3K, stimulating the phosphorylation of Akt, as has been shown in non-neuronal cell lines. It will be important to determine—in neuronal cultures—the causal relation between PI3K and Akt activation, and its downstream effectors, according to the particular 5-HT_1A_R distribution in neurons. Furthermore, in rat cortical primary cultures, it has been reported that 5-HT_1A_R activation promotes a destabilization of microtubules, reducing the transport of vesicles that contain the NR2B subunits of the NMDA receptor to dendrites and therefore, reducing channel conductance (Yuen et al., 2005). These evidences indicate that 5-HT_1A_R can regulate microtubule reorganization and both organelle and receptor trafficking.

## 5-HT_1A_R Forms Complex with GPCRs: A Mechanism to Modulate Its Signaling

Several reports have described that a wide variety of GPCRs expressed in recombinant cell systems may form homodimers and heterodimers. Some evidences suggest that GPCR dimer/oligomer species may differ in several aspects with the non-associated receptors, including ligand binding affinity and pharmacological profile, G-protein coupling, receptor trafficking and desensitization (Milligan, 2007). It has been described that 5-HT_1A_R constitutively forms homodimers in transfected HEK 293 cells; however the agonist favors the interaction of monomers, while the presence of antagonist reduces dimer formation (Łukasiewicz et al., 2007). Interestingly, the 5HT_1A_R may also form heterodimers with several GPCRs, creating new receptor species that may display a different behavior in comparison to individual receptors. For instance, stimulation of cells expressing either 5-HT_1A_R or mu-opioid receptors with specific agonists triggers in both cases, the activation of MAPK, cascade which desensitizes after 30 min of stimulation. Nonetheless, when both receptors are co-expressed, the activation of one receptor in the 5-HT_1A_R/μ-opioid heterodimer inhibits MAPK activation of the other receptor (Cussac et al., 2012). On the other hand, biochemical studies accomplished in neuroblastoma N1E-115 cells revealed that 5-HT_1A_R forms dimers and homo-oligomers, being dimers the prevalent species at the plasma membrane (Kobe et al., 2008; Woehler et al., 2009). Moreover, kinetics of 5-HT_1A_R dimer dissociation or association into high order homo-oligomers is not influenced by ligand binding (Kobe et al., 2008). For instance, the specific formation of 5-HT_1A_R-5-HT_7_R heterodimers was evidenced by co-immunoprecipitation and Forster resonance energy transfer (FRET) approaches in transfected N1E-115 cells with tagged-receptors (Renner et al., 2012). Furthermore, this study indicated that when both receptors are expressed in similar levels, the formation of 5-HT_1A_R-5-HT_7_R species is favored in comparison to the 5-HT_1A_R-5-HT_1A_R homodimer (Renner et al., 2012). Functional analyses using recombinant protein expression in *Xenopus* oocytes showed that co-expression of 5HT_1A_R and 5HT_7_R decreases 5-HT_1A_R-mediated activation of Gαi and GIRK channel activity, without affecting 5-HT_7_R mediated activation of Gs (Renner et al., 2012). This study also showed that both receptors are endogenously expressed in cultured hippocampal neurons and that after the knock-down of 5-HT_7_R with siRNA, GIRK activity is reduced by a 5-HT_1A_R agonist (Renner et al., 2012). This evidence, along with co-immunoprecipitation of both receptors in brain lysates (Renner et al., 2012), suggests a negative regulation of 5-HT_1A_R signaling driven by the presence of 5-HT_7_R. Moreover, the finding that during development 5HT_1A_R varies its expression and distribution (i.e., somato-dendritic shift; Patel and Zhou, 2005) and that 5-HT_7_R reduces its expression (Kobe et al., 2012), it is reasonable to think that, *in vivo*, there is a variation in the proportion of heterodimeric receptors, which may impact 5HT signaling mediated by the 5-HT_1A_R.

## Concluding Remarks

In summary, several studies have shown the coupling of 5-HT_1A_R with several signal transduction pathways in heterologous systems and only a few of these pathways have been studied in neuronal systems, where they are mainly associated with neuronal development, neuronal excitability and survival. Furthermore, it is likely that somatic receptors participate in the maintenance of neuronal survival, control gene expression and neuronal excitability. In contrast, those receptors located in dendrites would be more closely related to dendritic outgrowth and branching. Additional studies are needed to elucidate brain region- and neuronal-specific signaling mechanisms coupled to 5-HT_1A_R and their modulation by heterodimerization with other receptors, effects which may play a pivotal role in the actions of 5-HT during development and also, in some mood disorders.

## Author Contributions

PSR and JLF has written and edited the manuscript.

## Conflict of Interest Statement

The authors declare that the research was conducted in the absence of any commercial or financial relationships that could be construed as a potential conflict of interest.
